# Catastrophic health expenditures and economic burden of end-stage kidney disease among patients receiving hemodialysis or conservative care in Lao PDR

**DOI:** 10.1371/journal.pgph.0007532

**Published:** 2026-11-05

**Authors:** Mick Soukavong, Manit Sittimart, Thidachanh Soukhasem, Somvai Singhaxaiyaseng, Chanmaly Keomany, Oulai Oudommixay, Sysavanh Phommachanh, Elizabeth A. Ashley, Yot Teerawattananon, Mayfong MayxaY

**Affiliations:** 1 Unit for Health Evidence and Policy (UHEP), Institute of Research and Education Development, University of Health Sciences, Ministry of Health, Vientiane, Lao PDR; 2 Department of Natural and Social Sciences, Faculty of Medicine, University of Health Sciences, Vientiane, Lao PDR; 3 Health Intervention and Technology Assessment Program Foundation (HITAP), Nonthaburi, Thailand; 4 Department of Nephrology, Mittaphab Hospital, Vientiane, Lao PDR; 5 Microbiology Laboratory, Lao-Oxford Mahosot Hospital-Wellcome Trust Research Unit (LOMWRU), Mahosot Hospital, Vientiane, Lao PDR; 6 Nuffield Department of Medicine, Centre for Tropical Medicine and Global Health, University of Oxford, Oxford, United Kingdom; 7 Saw Swee Hock School of Public Health, National University of Singapore (NUS), Singapore, Singapore; University of Gothenburg: Goteborgs Universitet, SWEDEN

## Abstract

In Lao PDR, limited health insurance coverage poses significant challenges for patients requiring renal replacement therapy (RRT). However, the economic burden of end-stage kidney disease (ESKD), particularly for patients receiving hemodialysis compared with conservative care, remains poorly documented. We conducted a cross-sectional, prevalence-based, costing study, using consecutive sampling of ESKD patients receiving hemodialysis or conservative care at two tertiary hospitals in Lao PDR in 2025. A total of 117 patients were enrolled. Direct medical, direct non-medical, and caregiver productivity costs were collected through hospital surveys and patient interviews and annualized. National economic burden was extrapolated using estimated ESKD prevalence and assumed treatment distribution. The projected annual economic burden was estimated at 2.1 trillion kip (≈USD105 million) in 2025, based on an expert-informed treatment distribution used for national extrapolation. On average, this was approximately 280 million Kip/patient (≈USD13,692), largely driven by medical expenses. The average annual economic cost, comprised of medical, non-medical, and productivity costs, was 332 million Kip/year for hemodialysis patients, exceeding their average annual income (62 million Kip) by more than fivefold. Using 40% of total household income as an unadjusted proxy for capacity-to-pay, 83% of hemodialysis households and 63% of conservative care households exceeded the threshold, despite limited formal financial protection. Caregivers also experienced financial burdens through income loss and additional expenditures. Conservative care patients, though incurring lower costs, tended to be from economically vulnerable populations. ESKD imposes a considerable economic burden on patients and households in Lao PDR, particularly for those receiving hemodialysis in a setting with limited financial protection. These findings suggest that expanding health insurance coverage for ESKD patients and subsidizing non-medical costs are urgently needed.

## Introduction

End-stage kidney disease (ESKD) represents the final stage of chronic kidney disease (CKD), where kidney function is severely impaired and renal replacement therapy (RRT) is required to sustain life [1,2]. Globally, the burden of CKD and ESKD has been rising due to increasing prevalence of hypertension, diabetes, and population ageing. RRT options include hemodialysis (HD), peritoneal dialysis (PD), and kidney transplant [3]. While transplantation is the most effective long-term solution, hemodialysis is the most widely used form of RRT worldwide [4,5]. Despite advances in treatment, access to dialysis remains uneven, particularly in low- and middle-income countries (LMICs), where financial, infrastructural, and workforce constraints limit the availability of life-sustaining kidney care [6,7].

In the Lao People’s Democratic Republic (Lao PDR), the burden of ESKD is rising alongside the growing prevalence of non-communicable diseases [1]. Yet the country lacks a comprehensive system for early detection, diagnosis, and long-term management of CKD [2]. Dialysis services are limited to a small number of tertiary hospitals, mainly located in urban centers such as Vientiane. Geographic barriers, limited healthcare infrastructure, and shortages of trained personnel further restrict access to treatment. As a result, many patients are often presented at advanced stages of disease when treatment options are limited [2,7].

Financial protection for patients requiring dialysis in Lao PDR is also limited. Although some public insurance schemes provide partial subsidies for hemodialysis, coverage is inconsistent and often insufficient to meet the long-term needs of ESKD patients [2,6]. Consequently, many patients rely heavily on out-of-pocket (OOP) payments or family support. Some patients forgo dialysis and instead receive conservative care focused on symptom management rather than life-prolonging treatment [6,8]. While global studies have demonstrated the high economic burden associated with dialysis, particularly in LMIC settings, country-specific evidence is essential for informing health financing policies and resource allocation [9–13]. In Lao PDR, however, evidence gaps on the economic burden faced by ESKD patients remain.

The Lao government is currently considering expanding RRT under the National Health Insurance scheme [2]. However, without evidence on costs and financial risk, such expansion may be ineffective or inequitable. Particularly, little is known about the patient- and household-level economic burden of ESKD, including direct medical costs, non-medical costs, productivity losses, and the extent of catastrophic health expenditure. Furthermore, no previous study has estimated the national economic burden associated with ESKD using locally collected cost data. Addressing these evidence gaps is important for informing health financing policies and strengthening financial risk protection for patients requiring RRT. Therefore, this study aimed to estimate the annual economic burden of ESKD patients receiving hemodialysis or conservative care from a limited societal perspective, assess the proportion of catastrophic health expenditure among affected households, and estimate the national economic burden of ESKD in Lao PDR.

## Materials and methods

### Ethics statement

The study protocol was approved by the University of Health Sciences Research Ethics Committee, Ministry of Health, Lao PDR (Approval No. 886/REC). Written informed consent was obtained from all participants prior to interviews.

### Study design and setting

This cross-sectional study aimed to assess the economic burden of ESKD in Lao PDR. Adopting a limited societal perspective with the focus on patient and caregiver costs [14], primary data were collected over a four-month period (February–May 2025) from two major tertiary care hospitals in Vientiane—Mittaphab and Mahosot (conservative care costs only at this site as chronic hemodialysis services were not routinely available). These hospitals were selected due to their status as national referral centers within the national public health system for ESKD management. These hospitals receive referrals of patients with complex medical conditions, including chronic kidney disease, from provincial and district hospitals across the country. Mittaphab Hospital serves as one of the country’s principal providers of maintenance hemodialysis services, while Mahosot Hospital also manages patients with chronic kidney disease receiving conservative care and other specialist medical services.

### Study population

The study population included adults aged 18 years and above with a confirmed diagnosis of ESKD, who were receiving maintenance hemodialysis or conservative care at the participating hospitals during the study period. Notably, the exact number of current ESKD patients in Lao PDR was not readily available at the time this study was conducted. The estimated total number of ESKD patients was derived through stakeholder consultations for the overall economic burden calculation.

Inclusion criteria required an estimated glomerular filtration rate (eGFR) <7 ml/min/1.73m² without complications, or <15 ml/min/1.73m² with at least one complication as per the available CKD guideline in Lao PDR [15]. Patients must have received treatment for at least three months and be capable of providing informed consent directly or via a legal guardian. Exclusion criteria were: (i) diagnosis of acute kidney injury, (ii) receiving PD, (iii) history of kidney transplantation, (iv) incomplete medical records, or (v) inability to participate in interviews.

Sample size was calculated using a cost-of-illness sample size approach based on expected cost variability, with 10% precision [16], see also S1 Text. Based on a pilot of ten patients with ESKD attending Mittaphab hospital who met the study eligibility criteria, the standard deviation of monthly costs was approximately 2.5 million Kip. The pilot was conducted to assess question clarity, interview flow, and data completeness. To achieve 10% precision with 95% confidence, the required sample was 83 hemodialysis and 45 conservative care patients. Eligible patients were approached consecutively until the end of the study period.

This heterogeneous group may not represent a formal palliative or conservative kidney management program, but rather the current clinical reality in Lao PDR where patients remain on non-dialytic management due to resource, geographical, or personal constraints.

### Sampling method and recruitment

A consecutive sampling was used to recruit participants. Local nephrologists identified patients who met the predefined eligibility criteria at participating sites. Eligible patients (n = 131) were approached sequentially during routine clinical visits and invited to participate in the study. Patients receiving in-hospital hemodialysis or conservative care were eligible for recruitment. Patients receiving peritoneal dialysis were not included due to their extremely limited representation (fewer than five patients) in the country [C. Keomany, personal communication, August 27, 2024].

After receiving verbal and written information about the study, patients who agreed to participate provided informed consent. Each participant was assigned a unique identification number to maintain confidentiality and facilitate de-identification during data collection and analysis. If an eligible patient declined or was unavailable, the next eligible patient was approached. Face-to-face interviews with no recording, lasting approximately 40–60 minutes, were conducted by trained research assistants in Lao language. Notes were taken to capture the response from participants.

### Data collection

Cost-related data were obtained through primary data collection. This consisted of (i) a hospital survey for medical costs (e.g., unit cost and volume) relating to ESKD-related treatment services, specific to hemodialysis and conservative care and (ii) a structured interview questionnaire.

The questionnaire captured socio-demographic details, disease history, non-medical costs, opportunity costs, and healthcare utilization. Medical record reviews were performed by local nephrologists to check and extract clinical details and treatment modalities. For more details of the data collection forms, please see S2 Text.

### Variables and cost valuation

Costs were expressed in 2025 Lao Kip (approximately 20,444 Kip = 1 US$). A prevalence-based costing approach was applied [17], with a 12-month time horizon to reflect the annual economic burden. Given no national registry of ESKD patients exists in Laos, the national distribution of treatment modalities is unknown. For national extrapolation, the prevalence ratio of patients on hemodialysis and conservative treatment (expert opinions in stakeholder consultations) was assumed to be 30:70, for which different ratios were used in a sensitivity analysis. Historical costs were adjusted using the consumer price index (CPI). Cost valuation in this study followed these steps: identification of relevant resource use within the study period, measurement of the quantity of each resource consumed, and valuation of each resource using appropriate unit costs [18]. Cost estimation included three major categories [14,18].

*Direct Medical Costs* included diagnostic tests (e.g., labs, imaging), hemodialysis sessions, medications (e.g., erythropoietin, phosphate binders), hospitalizations, inpatient (IPD) and outpatient (OPD) costs, vascular access procedures, and treatment of complications. Where some medical items were presented as charges, they were converted into costs using a 0.8 cost-to-charge ratio (CCR). A CCR of 0.8 was applied based on available hospital administrative data for relevant treatment items provided by Mittaphab Hospital. However, facility-specific or service-specific CCRs were unavailable as detailed hospital cost-accounting data were not routinely collected for individual service lines. Therefore, a uniform CCR was applied across both study hospitals as a pragmatic approach. This value was also within a possible range of ratios in some LMIC tertiary hospitals [19]. The selected value was reviewed with local hospital administrators and nephrologists during stakeholder consultations and judged to be a reasonable approximation for the study setting. To assess the potential effect of this assumption, one-way sensitivity analyses varied the CCR from 0.4 to 1.0 to reflect the possibility that the base-case CCR of 0.8 may overestimate direct medical costs.

*Direct Non-Medical Costs* covered actual expenses incurred by patients and relatives from their transport to health facilities, food and accommodation (if applicable), caregiver expenses, and renal-specific dietary needs.

*Opportunity Costs* captured productivity losses using the human capital approach [20]. This approach was selected because it aligns better with our study objective, which is to understand household economic burden, not employer productivity loss or replacement costs. Under this approach, productivity losses were valued based on the income associated with time lost from paid work. The human capital approach was also considered appropriate and feasible in this setting because it is relatively transparent and requires less detailed data than approaches such as the friction cost approach, which may require information on labor-market replacement and the duration of productivity losses [20]. However, reliance on self-reported income may not capture the full extent of productivity losses, particularly unpaid work and informal household activities.

These included income loss due to work absenteeism among patients, as well as their relatives or informal caregivers (e.g., time spent accompanying or supporting the patient). Time lost was first measured using the past-week absenteeism from the Work Productivity and Activity Impairment Questionnaire: General Health V2.0 (WPAI-GH), which uses a 7-day recall period [21]. Specifically, respondents reported (i) hours missed due to health problems and (ii) hours actually worked during the previous week. The weekly absenteeism score was then calculated as: (i)/[(i) + (ii)]. This was then multiplied by expected weekly income to estimate the monetary cost of absenteeism. Expected weekly income was calculated by multiplying self-reported monthly income by 12 and divided by 52, as standardized national wage data for Lao PDR were not available. Weekly productivity losses were extrapolated to annual estimates by multiplying by 52.

All costs were extrapolated to annual estimates to allow comparison across treatment groups. Where data for specific events were available, these were annualized based on reported frequency of such events over the past 12 months. See also S3 Text for detailed relevant variables and unit costs.

### Quality assurance measures

Comprehensive quality control measures were implemented throughout the study. Research assistants were trained on data collection procedures to ensure consistency and accuracy. Pre-testing of all data collection instruments was conducted with ten patients to identify and address any potential issues before full implementation. Regular supervision and quality checks were performed by local nephrologists throughout the data collection period to maintain high standards. Double data entry was conducted for 10% of questionnaires to assess data quality and identify potential errors. When high variation was detected, a review of discrepancies was performed to verify and correct errors. Key stakeholders (e.g., local nephrologists and representatives from ESKD patient group, university research institutes, the Department of Healthcare and Rehabilitation, the Food and Drug Department, and public payers) were consulted during study design to ensure that relevant cost items and resource uses were comprehensively captured.

### Data analysis

Descriptive analysis was used to summarize patient characteristics and cost data. Continuous variables were reported as means with SD. Categorical variables were summarized as frequencies and percentages. Cost outcomes were presented as estimated mean value.

Sensitivity analysis involved one-way analyses to examine the extent to which total cost estimates are affected by the choice of parameters and method used in the estimations [22]. Parameters varied included the cost-to-charge ratio (CCR), the inclusion of specific items such as home modifications or assistive equipment, and the prevalence of ESKD patients (±10% and ±20%). Considering hemodialysis utilization from other settings which was often less than 30% as assumed in this study [23], variation in hemodialysis-to-conservative care ratios (10:90, 20:80, and 40:60) was also examined.

## Results

### Patient characteristics and sociodemographic profile

Out of 128 required samples, 117 patients provided informed consent and were able to participate in the study. Of these, 90 patients were receiving hemodialysis and 27 were receiving conservative care. The conservative-care group was smaller than the planned sample size of 45 participants, which reduced the statistical power and precision of estimates for this group. Table 1 presents a summary of patient characteristics. The average age of patients was 58 years, with an approximately equal gender distribution. The majority were married (68%) and had not attained undergraduate-level education (68%).

**Table 1:** Baseline characteristics of patients with ESKD involved in the study.

Particular/Characteristic	Conservative Care (N = 27)	Hemodialysis (HD) (N = 90)	ESKD Patient (Total N = 117)
**Gender Female/male (N)**	12/15	46/44	58/59
**Average Age Years (SD)**	62 (15)	57 (14)	58 (14)
**Education level (%)**			
Below undergrad degree	78	64	68
Undergrad degree	18	22	20
Higher than undergrad	–	13	10
Other (informal education)	4	1	2
**Marital status (%)**			
Single	8	6	6
Married	59	71	68
Divorced	11	2	4
Spouse deceased	22	20	21
Other	–	1	1
**Income level (Kip/Month)**			
Average among ESKD patients (SD)	1,757,692 (2,214,797)	5,217,361 (16,346,020)	4,392,119 (14,896,535)
Median value (IQR)	1,200,000 (2,350,000)	2,150,000 (3,500,000)	2,200,000 (3,500,000)
Source of income to support treatment* (%)			
Government support (e.g., insurance and welfare)^#^	9	23	20
OOP - personal and family support	87	64	68
Sale assets, property, loans	4	13	12
^**#**^ **Coverage among different health insurance/government support (%)**			
General health insurance	–	–	–
Government employee scheme	15	32	30
Social Security scheme	15	34	32
State enterprise scheme	8	10	9
Private health insurance	–	2	2
Others (e.g., community health insurance, soldier insurance)	62	22	27
Common Symptoms/Comorbidities observed* (%)			
High blood pressure (hypertension)	50	46	47
Diabetes (Type-2)	15	23	22
Stroke	2	2	2
Gallbladder stones	9	8	9
Ischemic heart disease	18	6	8
Other (e.g., gout, lung disease, liver disease, rheumatoid arthritis, Benign prostatic hyperplasia, Systemic lupus erythematosus, Thalassemia, Thyroid, Seizure)	6	15	12

CI = Confidence Interval; *One person may be in multiple groups; ^#^Applicable only to those who identified government support as one of their sources to support treatment; N/A = not available; Data were presented either as mean value (SD) or proportion; All costs are reported in 2025 Lao Kip; Calendar Year Average Consumer Price Index = 251.39; IQR = Interquartile range.

Among the samples, most ESKD patients (77%) were receiving hemodialysis, while 23% relied on conservative care. For hemodialysis, patients underwent dialysis approximately 2 sessions per week. Among patients currently receiving hemodialysis, 71% reported having previously undergone conservative care for an average of three months before initiating dialysis, whereas 29% reported initiating hemodialysis without a preceding period of conservative care. Patients in the current conservative care group had not previously received hemodialysis.

### Health insurance, financial coping, and economic status

Approximately 20% of patients were covered or partially supported by government or social security schemes. Although some patients reported coverage from multiple schemes, this did not equate to adequate financial protection. Noteworthy, a greater proportion of patients with hemodialysis were found to have insurance coverage, compared to that of patients with conservative care.

A notable 68% of recruited patients paid out-of-pocket (OOP), with household income identified as the primary funding source for overall ESKD management. While many relied heavily on personal savings and family support, it is important to note that additional coping strategies including the sale of assets and borrowing funds were also exhibited. A greater proportion of patients relying on these coping mechanisms were found among the hemodialysis group, given a higher overall maintenance cost for hemodialysis.

The average monthly income was about 4.4 million Kip across ESKD patients, though this was highly skewed, with a maximum reported income of 150 million Kip. Patients managed through conservative care had substantially lower average incomes (~1.8 million Kip) compared to those undergoing hemodialysis (~5.2 million Kip).

### Economic cost of ESKD

Applying the expert-informed 30:70 HD-to-conservative care ratio for national extrapolation, the projected annual economic burden of ESKD in 2025 was estimated at 2.1 trillion kip. Direct medical costs constituted the largest share of the total burden (69%), amounting to 1,486,104 million kip. Direct non-medical costs and opportunity costs each accounted for approximately 15–16% of the total (Table 2).

**Table 2:** Summary of economic burden of ESKD in Lao PDR, 2025.

Population	Cost category	Estimated annual economic cost (expressed in million kip)	Percentage (%)
*Overall ESKD patients*	Total economic cost of ESKD*	2,145,823	100
	Direct medical costs	1,486,104	69
	Direct non-medical costs	312,367	15
	Opportunity costs paid by households	347,351	16
	Average economic cost per patient [95% CI]	280 [170-390]	
*ESKD patients under conservative care*	Total economic cost among conservative care patients*	1,383,711	100
	Direct medical costs	1,004,728	73
	Direct non-medical costs	165,575	12
	Opportunity costs paid by households	213,409	15
	Average economic cost per conservative care patient [95% CI]	258 [157 – 359]	
*ESKD patient under hemodialysis*	Total economic cost among hemodialysis patients*	762,126	100
	Direct medical costs	481,380	63
	Direct non-medical costs	146,799	19
	Opportunity costs paid by ESKD households	133,947	18
	Average economic cost per HD patient [95% CI]	332 [201-461]	

^*^Population estimates were based on stakeholder-informed assumptions because a national ESKD registry is not available. The estimates were derived, assuming approximately 7,666 patients with ESKD nationally. Under the base-case assumption that 30% received maintenance hemodialysis and 70% received conservative care, the corresponding populations were estimated as 2,300 patients receiving hemodialysis (30%) and 5,366 patients receiving conservative care (70%); All costs are reported in 2025 Lao Kip. USD equivalents were calculated using the average 2025 official exchange rate (20,444 Kip = 1 US$) and are presented for international comparison only. Calendar Year Average Consumer Price Index = 251.39; 95% CI = 95% Confidence Interval, estimated based on normal approximation method.

On average, the annual economic burden per ESKD patient was 280 million kip (with OOP costs at roughly 235 million kip). This substantially exceeded the estimated average annual ESKD patient income (approximately 52 million kip), indicating considerable financial strain at the household level. Following the WHO approach to catastrophic health expenditure (CHE), which defines CHE as OOP payments ≥40% of a household’s capacity to pay [24], we assessed CHE prevalence. As per WHO methodology, capacity-to-pay ideally represents income after deducting essential subsistence expenditures (typically food costs) [25]. However, given the unavailability of detailed household expenditure data in our study setting, we approximated capacity-to-pay using total annual household income. This approach may overestimate true capacity-to-pay and thus potentially underestimate CHE prevalence. Nonetheless, using this approximation, 83% of hemodialysis households and 63% of conservative care households exceeded the threshold.

National aggregate costs were high for conservative care, reflecting the larger proportion of patients assumed to receive conservative management compared with hemodialysis. However, at the individual level, the average annual economic cost per patient was higher for hemodialysis (332 million kip) than for conservative care (258 million kip), reflecting the greater resource intensity of hemodialysis treatment. Estimates for the conservative-care group should be interpreted with caution, given that the group had fewer participants than originally planned, potentially resulting in lower statistical precision.

Across both treatment modalities, direct medical costs were the dominant component of total economic costs. Nevertheless, the composition of non-medical costs differed. Among households of patients receiving conservative care, opportunity costs represented a larger proportion of total costs than direct non-medical costs. In contrast, among households of hemodialysis patients, direct non-medical costs constituted a relatively greater share of total costs than opportunity costs.

### Sensitivity analysis

As shown in Table 3, sensitivity analyses demonstrated that the estimates of ESKD economic burden in 2025 could range from 1.6 trillion kip (-27% change from the baseline) to 2.6 trillion kip (+20% change from baseline). Among scenarios and parameter choices examined, the estimated prevalence of ESKD was one of the key drivers, impacting the total economic burden. This finding is expected, as prevalence directly determines the size of the affected population and therefore proportionally scales total costs. The CCR was also another driver of variation, given that direct medical costs had the largest share of total economic burden. In addition to that, changes in the distribution of treatment modalities influenced total estimates. An increased proportion of patients receiving hemodialysis resulted in higher overall economic burden, given the greater average annual cost per hemodialysis patient compared with conservative care.

**Table 3:** Summary of variation of economic cost of ESKD from sensitivity analysis.

**Baseline**	2,145,823	Reference
**S1: ESKD Prevalence in 2025** *(Baseline value: 7,666)*		
S1.1 Prevalence increased from baseline by 10%	2,360,405	+10
S1.2 Prevalence increased from baseline by 20%	**2,574,987**	**+20**
S1.3 Prevalence decreased from baseline by 10%	1,931,240	-10
S1.4 Prevalence decreased from baseline by 20%	1,716,658	-20
**S2: Ratio of hemodialysis (HD): Conservative Care in the prevalence** *(Baseline value: 30/70)*		
S2.1 Ratio of 10 HD:90 Conservative Care	2,033,143	-5
S2.2 Ratio of 20 HD:80 Conservative Care	2,089,483	-3
S2.3 Ratio of 40 HD:60 Conservative Care	2,202,162	+3
**S3: Cost-to-charge ratio (CCR) for related medical costs** *(Baseline value: 0.8 CCR)*		
S3.1 Decreased CCR to 0.4	**1,558,625**	**-27**
S3.2 Decreased CCR to 0.5	1,705,424	-21
S3.3 Decreased CCR to 0.7	1,999,023	-7
S3.4 Increased CCR to 0.9	2,292,622	+7
S3.5 Increased CCR to 1 (no markup)	2,439,422	+14
**S4: Consideration of assistive equipment and house modification costs** *(Baseline: no inclusion)*		
S4.1 Included both costs	2,356,156	+10
S4.2 Included assistive equipment only	2,322,878	+8

S=Scenario; All costs are reported in 2025 Lao Kip; Calendar Year Average Consumer Price Index = 251.39.

## Discussion

This study demonstrates a substantial economic burden of ESKD in Lao PDR, driven primarily by high OOP costs among patients receiving hemodialysis. In the context of limited health insurance coverage, many patients bear OOP expenses that exceed their reported income, placing them at considerable risk of catastrophic health expenditure. Despite this, only a minority of participants reported coverage through formal health financing mechanisms, highlighting serious gaps in financial-risk protection.

The observed differences in economic burden across treatment modalities raise important concerns regarding equity in access and affordability. Although patients receiving conservative care incurred lower direct medical costs, they also had markedly lower incomes than their hemodialysis counterparts. This difference is unlikely to reflect an effect of treatment modality itself but more plausibly indicates socioeconomic selection into treatment pathways. Households with greater financial resources may be more likely to initiate and sustain hemodialysis despite its substantial out-of-pocket costs, whereas patients with fewer resources may rely on conservative care because of affordability constraints, geographical barriers, or limited-service availability. Consequently, comparisons of economic costs between treatment groups should be interpreted as descriptive rather than causal. Noteworthy, the economic burden of ESKD is likely to be regressive, disproportionately affecting poorer households [2,26].

Beyond direct costs, both non-medical and opportunity costs further compound the burden on patients and their households. Caregivers experience income loss alongside additional expenditures for transportation, food, and home adjustments. These findings convey the importance of adopting a broader societal perspective when assessing the economic impact of ESKD, as those costs are often overlooked in policy discussions despite their substantial contribution to overall burden [27,28].

Although evidence from Lao PDR has been scarce, our findings are consistent with regional and global studies demonstrating the high cost of dialysis, particularly in settings with fragmented or limited insurance coverage [29–31]. The projected burden of ESKD in Laos was equivalent to approximately 0.5% of the country’s estimated nominal GDP in 2025, based on GDP estimates reported by the International Monetary Fund (IMF) [32]. This proportion is comparable to estimates reported in Malaysia [33]. However, this is higher than those observed in settings such as Iran and Italy (approximately 0.3% and 0.11% of GDP, respectively) [34,35]. Cross-country comparisons should nevertheless be interpreted cautiously. This is due to the variations in the pricing structures (public procurement versus private provision), the scope of included cost components, contextual factors, and the methodological variances to cost estimation. Furthermore, many studies reported costs as a proportion of total health expenditure rather than GDP, limiting direct comparability.

These findings have several significant implications for health financing, renal care delivery, and CKD care improvement in Laos. Firstly, reducing OOP payments for hemodialysis should be considered to strengthen financial risk protection and prevent catastrophic expenditure [1]. Secondly, the observed inequities between treatment modalities suggest the need to ensure access to RRT is guided by clinical need rather than ability to pay. This is particularly relevant as Lao PDR considers expanding access to kidney care services [2]. Thirdly, capturing and integrating indirect and opportunity costs into policy discussions may support more realistic assessments of the full economic impact of ESKD on households. Finally, given the substantial resource requirements associated with RRT, strengthening conservative care and early-stage CKD management, including enhancing patient awareness and clinical follow-up, should be considered alongside broader strategies. This might help delay progression to ESKD, thereby potentially reducing future long-term demand for RRT, alleviating pressure on the health system and moderate overall economic burden [36]. Such preventive strategies could also become vital for rural communities [37]. As these strategies are implemented, robust monitoring systems should track both clinical outcomes and household financial burden to ensure equitable access and financial risk protection.

This study has several limitations. Firstly, the analysis was based on patients recruited through consecutive sampling from two tertiary hospitals in Vientiane, which may introduce selection bias and thereby limit generalizability to the broader ESKD population in Lao PDR, particularly in rural areas where access to dialysis is more constrained. As a result, our estimates likely underestimate true economic burden for patients traveling long distances. Also, the sample size achieved for the conservative care group was smaller than originally planned. Consequently, the estimated costs for this group are subject to greater sampling variability and lower statistical precision than anticipated. Secondly, national economic burden estimates relied on assumptions regarding the prevalence of ESKD and treatment distribution ratio, given the absence of a national kidney disease registry. The treatment distribution observed in our hospital-based sample (77% receiving hemodialysis and 23% receiving conservative care) should therefore not be interpreted as representative of the national ESKD population. This distribution may have overrepresented patients receiving hemodialysis because participants were recruited from two tertiary referral hospitals where hemodialysis services are available. In contrast, patients with ESKD who are unable to initiate or sustain hemodialysis because of limited-service availability, financial constraints, or geographical barriers may remain on conservative management or may not reach tertiary dialysis centers and would therefore be less likely to be captured in our sample. For national extrapolation, we therefore used an expert-informed 30:70 ratio of hemodialysis to conservative care, based on consultations with local nephrologists and key stakeholders, as a projection of the broader national treatment distribution rather than an estimate derived from the study sample. Nevertheless, in the absence of nationally representative data, this treatment distribution remains uncertain, and the effect of alternative treatment ratios on the estimated economic burden was examined through sensitivity analyses. The national burden estimates should be interpreted as approximate rather than definitive. Thirdly, income, expenditure, and some cost data were self-reported and may be subject to social desirability or recall bias, particularly given the sensitive nature of financial hardship. Estimation of productivity loss, using a 7-day recall period from the WPAI-GH questionnaire, could also potentially introduce additional recall and extrapolation bias, particularly given the fluctuating nature of ESKD-related symptoms and complications. Fourthly, the analysis focused on hemodialysis and conservative care and did not include other modalities such as peritoneal dialysis or kidney transplantation, which may underestimate the full economic burden of ESKD and preclude a comparison across all RRT options. Also, the use of a uniform CCR may not fully capture variation across facilities and service categories, and that the projected base-case economic burden is sensitive to this assumption. Fifthly, the Lao Kip depreciated by approximately 30% during 2024–2025, affecting the USD conversion; readers should interpret dollar figures cautiously. In the assessment of CHE prevalence, as detailed household expenditure data were unavailable, capacity-to-pay was approximated using total annual household income. Therefore, the reported proportions should be interpreted as unadjusted household-income-based estimates using the 40% threshold rather than standard WHO CHE estimates, and this may underestimate the true CHE prevalence. Finally, as this was a prevalence-based cross-sectional design study, only patients alive and receiving treatment during the study period were included. Consequently, the estimated annual costs primarily represent individuals surviving with ESKD rather than those who died shortly after treatment initiation or experienced rapid disease progression. This survivor effect may influence the representativeness of average per-patient costs, although the direction of any bias is uncertain. Patients with early mortality may incur substantial acute care costs before death, whereas longer-term survivors accumulate ongoing dialysis and follow-up costs over time. Future incidence-based or longitudinal studies would better capture lifetime costs across the full disease trajectory.

Peritoneal dialysis and kidney transplantation were excluded due to their currently low utilization. Although growing, kidney transplantation services remain relatively new and were performed with overseas assistance. As these modalities expand, future research should incorporate their costs and assess their comparative cost-effectiveness. Implementation research is also needed to explore the feasibility, accessibility, and acceptability of expanding RRT services. This should include assessments of healthcare workforce capacity, infrastructure readiness, and system-level constraints. Such evidence will be critical to inform the equitable and sustainable development of kidney care services in Lao PDR.

## Conclusions

This study provides critical insights into the projected annual economic burden of ESKD treatment in Lao PDR, emphasizing the substantial OOP costs associated with hemodialysis and the inequities in access to RRT. Our findings reveal that many patients, particularly those receiving hemodialysis, had OOP healthcare costs that exceeded the specific threshold relative to their reported income, with limited protection from existing health financing schemes, suggesting a considerable risk of financial hardship. The economic impact extends beyond patients to their families and caregivers, compounding the overall burden.

Although the magnitude of the burden should be interpreted with appropriate caution, the study highlights an urgent need for policy interventions to strengthen financial risk protection, improve equitable access to kidney care services, and reduce the economic burden borne by vulnerable households. Strengthening the prevention and early management of CKD, together with improving national insurance coverage and developing mechanisms to better address non-medical costs associated with treatment, will be important for reducing disparities while supporting a more sustainable kidney care system. Further research and implementation studies are needed to identify context-appropriate strategies for expanding equitable kidney care in Lao PDR.

## Supporting information

S1 Text Sample size calculation and participant sampling process. (PDF)

S2 Text Data collection tools. (PDF)

S3 Text Detailed items and unit costs. (PDF)

S1 STROBE STROBE Checklist. (PDF)
