# Supplementary material for: Catastrophic health expenditures and economic burden of end-stage kidney disease among patients receiving hemodialysis or conservative care in Lao PDR
Source: PLOS Glob Public Health. 2026 Nov 5;6(11):e0007532. doi: 10.1371/journal.pgph.0007532 (PMC13739169; doi:10.1371/journal.pgph.0007532)
Supplement: Supplementary file 1 [file pgph.0007532.s001.pdf]

Supplementary files

Supplementary 1: Sample size calculation and participant sampling process

Sample size calculation

The sample size required to estimate costs of hemodialysis (HD) and conservative care was calculated using the coefficient of variation approach for cost studies, as follows:

$$n = (1.96 \times \frac{Cv}{P})^2$$

where:

- *n* = required sample size;
- *Cv* = coefficient of variation;
- *P* = desired relative precision (10%);
- 1.96 corresponds to the 95% confidence level.

To account for an anticipated 10% non-response or incomplete data rate, the calculated sample size was inflated using: $N = (n + \frac{n}{10})$

Table 1 Summary of sample size calculation based on Cv, Precision and non-compliance

N	Co-efficient of variation for HD	Co-efficient of variation for Conservative	Precision (%)	sample size, including non-compliance (10%) for HD	sample size, including non-compliance (10%) for Conservative care	Total sample size, including non-compliance (10%)
1.	0.44*	0.33*	10	83	45	128

**Co-efficient of variation (Cv) was derived from the information (mean and standard deviation of the costs of RRT) provided in the previous study conducted in Thailand, as no comparable cost data were available from Lao PDR during the study planning stage.*

Total required sample size (including 10% allowance for non-response): 128 participants.

Participant Sampling

A simple random sampling approach was used to recruit participants from the eligible patient populations of the participating tertiary hospitals. The study population comprised of adult patients with end-stage kidney disease (ESKD) receiving either HD or conservative care during the study period.

Inclusion criteria

Patients were eligible if they:

- had ESKD, defined as an estimated glomerular filtration rate (eGFR) <7 mL/min/1.73 m^2 without complications, or <15 mL/min/1.73 m^2 with at least one complication according to the Lao PDR chronic kidney disease guideline;
- had received HD or conservative care for at least three months;
- were aged 18 years or older; and
- were able to provide written informed consent independently or through a legally authorized representative.

Exclusion criteria

Patients were excluded if they:

- had acute kidney injury;
- were receiving peritoneal dialysis;
- had previously undergone kidney transplantation;
- had incomplete medical records required for cost estimation; or
- were unable to participate in the interview because of severe illness, cognitive impairment, or communication barriers.

Participant Selection

Lists of eligible patients were obtained from hospital renal unit records after screening against the eligibility criteria by local nephrologists. Each eligible patient was assigned a unique study identification number. Separate sampling frames were prepared for HD and conservative care patients. Participants were then selected using a computer-generated random number sequence to ensure that every eligible individual had an equal probability of selection.

Selected patients were approached during routine clinical visits. After receiving verbal and written information about the study, patients who agreed to participate provided written informed consent. If a selected patient declined participation or was unavailable during the recruitment period, another participant was randomly selected from the remaining eligible patients using the same random sampling procedure until the target sample size was achieved.

It should be noted that conservative care patients recruited were those managed without maintenance hemodialysis under routine clinical practice. This group was not restricted to patients enrolled in a formal conservative kidney management or palliative care program. Rather, conservative care reflected the existing care pathway, in which patients remained on non-dialytic treatment for a variety of reasons, including financial constraints, geographical barriers, limited dialysis availability, patient preference, or clinical considerations.

Data Collection Procedures

a. Training of Research Staff

Research assistants received training on study objectives, participant recruitment, informed consent procedures, interview techniques, questionnaire administration, and data management to ensure consistency across study sites.

Prior to the main study, all data collection instruments were pilot tested with ten ESKD patients who were not included in the final analysis. The pilot participants were selected purposively to reflect both treatment modalities and to evaluate the clarity, comprehensibility, and feasibility of the questionnaires and data collection procedures. Feedback from the pilot study was used to refine question wording, improve interview flow, and standardize data collection procedures before field implementation.

Throughout data collection, local nephrologists provided regular supervision and reviewed all completed questionnaires to ensure adherence to the study protocol.

b. Recruitment and Informed Consent

Selected participants were approached by trained research staff and provided with detailed information regarding the study objectives, procedures, potential risks, and expected benefits. Written informed consent was obtained before enrolment. All study procedures complied with the approved ethical protocols, and participant confidentiality was maintained through anonymization of all study records.

c. Data Collection

Direct medical cost data were extracted from hospital billing systems, financial records, and patients' medical records. A hospital survey was also launched for data triangulation. Information on direct non-medical costs and opportunity costs was collected through structured face-to-face interviews with patients. When appropriate, accompanying family members or informal caregivers also contributed information regarding caregiving time and related expenditures.

d. Data Management and Quality Assurance

Completed questionnaires were entered into a Microsoft Excel database by trained research assistants. To ensure data accuracy, double data entry was performed for a randomly selected 10% sample of questionnaires. Discrepancies identified during verification were resolved through comparison with the original data collection forms. Additional consistency checks and periodic quality reviews were conducted throughout the study to minimize data entry errors and maintain data integrity.

Sources:

Teerawattananon, Y., M. Mugford, and V. Tangcharoensathien, *Economic Evaluation of Palliative Management versus Peritoneal Dialysis and Hemodialysis for End-Stage Renal Disease: Evidence for Coverage Decisions in Thailand.* Value in Health, 2007. 10(1): p. 61-72.

Pandya, S. *Recommendations on care and management of Chronic Kidney Disease (CKD)*. Chapter 10: Chronic Kidney Disease: causes 2016 [cited 2024 August 20]; Available from: https://kidneyeducation.com/Lao/

Althubaiti A. *Sample size determination: A practical guide for health researchers*. *J Gen Fam Med*. 2023; 24: 72–78. https://doi.org/10.1002/jgf2.600
