# Supplementary material for: Catastrophic health expenditures and economic burden of end-stage kidney disease among patients receiving hemodialysis or conservative care in Lao PDR
Source: PLOS Glob Public Health. 2026 Nov 5;6(11):e0007532. doi: 10.1371/journal.pgph.0007532 (PMC13739169; doi:10.1371/journal.pgph.0007532)
Supplement: Supplementary file 2 [file pgph.0007532.s002.pdf]

Supplementary files

Supplementary 2: Data collection tools

Survey Form for Direct Medical Cost Data for Treatment of End-Stage Kidney Disease (ESKD): Conservative Care and HD [translated version]

This hospital survey form is part of research collaboration between the Unit for Health Evidence and Policy (UHEP), University of Health Sciences (UHS) Lao PDR; the Lao-Oxford-Mahosot Hospital-Wellcome Trust Research Unit (LOMWRU); and the Health Intervention and Technology Assessment Program Foundation (HITAP), Thailand.

Research Project: Economic Evaluation of Renal Replacement Therapy (RRT) for End Stage Kidney Disease in Lao PDR.
Research Objectives: To provide economic and feasibility evidence for RRT for patients with ESKD in Lao PDR.

Date of data collection:…………………………………………………………………………….

Collection site:……………………………………………………………………………… *[name of hospital/department]*

Completed by:…………………………………………………………………….*[name or contact for further validation]*

N	COST TYPE/ITEM	UNIT	COST/UNIT	SOURCE
	Registration Book (1st time only)			
	Doctor fee			
I	**Conservative care**			
	medicine (kip/month)			
	Medication: ORAL			
	Medication: INJECTION			
	Lab test (please specify)			
	Echo Abdo			
	X-ray Abdo			
	CT scan Abdo			
	Complication treatment (Please specify in section IV if possible)			
	Any other relevant costs (please specify)			
II	**PD**			
	Cost of catheter implantation			
	Cost of dialysis solution with bag (per set)			
	Lab test (please specify)			

N	COST TYPE/ITEM	UNIT	COST/UNIT	SOURCE
	Echo Abdo			
	X-ray Abdo			
	CT scan Abdo			
	Complication treatment (Please specify in section IV if possible)			
	Any other relevant costs (please specify)			
III	**HD**			
	Lab test (please specify)			
	Echo Abdo			
	X-ray Abdo			
	CT scan Abdo			
	Cost of vascular access			
	Unit cost of HD			
	Dialysis sessions per patient per week			
	Complication treatment (Please specify in section IV if possible)			
	Any other relevant costs (please specify)			
IV	**Cost of Complication**			
	Cost of treating peritonitis			
	Average cost of treatment peritonitis			
	Average cost of treatment vascular access related conditions			
	Average cost of conservative care complications			
	Any other relevant costs (please specify)			

Contact of focal points in case of doubt

Dr Mick Soukavong (micksoukavong@gmail.com) or
Dr Thidachanh Soukhasem (thidachanh_pp@hotmail.com)
Unit for Health Evidence and Policy (UHEP), Lao PDR

The research team would like to thank you for giving us the opportunity to further examine this study.

Patient and Household Survey [translated version]

Economic Evaluation of Renal Replacement Therapy (RRT) for End Stage Kidney Disease (ESKD)

In collaboration with the Unit for Health Evidence and Policy (UHEP), University of Health Sciences (UHS) Lao PDR, the Lao-Oxford- Mahosot Hospital- Wellcome Trust Research Unit (LOMWRU) and the Health Intervention and Technology Assessment Program Foundation (HITAP), Thailand.

Survey : Basic information, medical history, cost of illness and quality of life of patients with end-stage renal disease for renal replacement therapy.

Origin and purpose : To investigate the cost of illness and assess the general health-related quality of life of patients with end-stage renal disease treated with renal replacement therapy.

Uses of the results from this survey : The results will be used to assess the cost-utility of renal replacement therapy (RRT) in the Lao population, and to analyze the budget impacts, in order to develop recommendations for the development of a future national health benefits package. The researchers will not disclose any personally identifiable or sensitive information to the public. The data will be used only for this research , and will be presented to stakeholder meetings and policymakers for future policy decision- making .

This survey is divided into 3 parts as follows:

Part 1 General information Demographic information
Part 2 Treatment and illness information (Clinical-demographic profile)
Part 3 Patient expenses (Direct non-medical cost and indirect cost)

Person and method of contact in case of doubt

Contact of focal points in case of doubt
Dr Mick Soukavong (micksoukavong@gmail.com) or
Dr Thidachanh Soukhasem (thidachanh_pp@hotmail.com)
Unit for Health Evidence and Policy (UHEP), Lao PDR

The researchers would like to thank everyone who took the time to provide useful information for this study

Questionnaire

***For* collecting basic information , medical history, cost of illness and quality of life in patients with end stage renal disease (End Stage Kidney Disease: ESKD) for Renal Replacement Therapy (RRT) procedures.**

Statement

1. This data collection form consists of 4 sections, with 13 main pages. Please check the completeness of the pages before starting to fill in the information.
2. Fill in the information using a black or blue ballpoint pen only.
3. When filling in the squares, fill in the center of the box. Do not write outside the square box.
   - Filling in the squares ❑is used to select the desired item by marking the ✓item in front of it. In case of editing, **do not delete** , but double-draw.~~✓~~ And sign your name with the date clearly .
   - Filling in the squares ☐is used to fill in numbers in that box.
     - If there is no number to fill in the blank, fill in the number 0 in the empty square (do not leave a space).
     - In the case of numbers in the thousands or more, do not enter the "," symbol. For example , 1,200 , enter as [0][0][1][2][0][0]
     - In case of correction, do **not delete.** Draw a line across the double ~~2~~and clearly state your name and date. Then write the correct number outside the box where the incorrect information is entered.

***** This information is anonymous and will be kept confidential for use in this research only.***

To benefit the development of the future health service system**

Please mark ✓the square or circle and fill in the blanks.

day month year (B.E.) Data Collection Date ☐☐/ ☐☐/☐☐☐☐

ID: ☐☐☐

Name of data collector ....(Please state your full name clearly)

Name of hospital where data is collected

❑ (1) Mittaphap Hospital ❑ (2) Hospital [ ]

❑ (3) Hospital [ ]

Part 1 General information of the patient (current)

1. sex ❑ (1) Male ❑ (2) Female
2. Age ....years
3. Family status

   ❑ (1) Single ❑ (2) Married ❑ (3) Divorced

   ❑ (4) Living apart (e.g. due to spouse's death) ❑ (5) Other, please specify...............................
4. Main rights to medical treatment

   ❑ (1) Universal health insurance ❑ (2) Civil servants

   ❑ (3) Social security ❑ (4) State enterprises

❑ (5) Private health insurance ❑ (6) Pay by yourself

❑ (7) Other, please specify.....................................

5. Level of education of the patient

❑ (1) Below bachelor's degree level

❑ (2) Bachelor's degree

❑ (3) Higher than bachelor's degree level

❑ (4) Others, please specify ....(e.g. studying in a vocational field or not studying at a level, etc.)

6. Family income **per month** ....Kip

Part 2 Disease and treatment information

7. When did you start undergoing renal replacement therapy (RRT)?

day month year (B.E.) ☐☐/ ☐☐/☐☐☐☐

8. Please tick the appropriate box based on renal replacement modality patient is currently on.
❑ Hemodialysis (HD) *(proceed to 9)*
❑ Peritoneal Dialysis (PD) *(proceed to 10)*
❑Conservative Care *(proceed to 11)*

9. **FOR HEMODIALYSIS:** How many sessions of hemodialysis do you undergo in a week?

....................................Sessions/WEEK

How much time (in hours), on average, do you spend for each session of dialysis at your dialysis center/hospital?

....................................Hours/SESSION

10. **FOR PERITONEAL DIALYSIS:** How many PD exchanges do you have in a day?

....................................Exchanges/DAY

How much time do you spend (hours) changing the dialysate at home?

....................................Hours/EXCHANGE

How frequently do you have to go to a hospital to collect PD exchange fluid?

....................................times/WEEK

11. **FOR CONSERVATIVE MANAGMENT:** What treatments are currently provided as part of your RRT? (if many, please list them up to five ongoing treatments)

....
....
....
....
....

12. **ASK QUESTION BASED ON MODALITY PATIENT IS CURRENTLY ON AND INDICATE DURATION IN PREVIOUS MODALITY:**
IF DIALYSIS: Were you on PD/HD before?

IF CONSERVATIVE MANAGMENT: Were you on dialysis before?

- ❑ No switch
- ❑ PD to HD
- ❑ HD to PD
- ❑ PD to Conservative management
- ❑ HD to Conservative management
- ❑ Conservative management to PD
- ❑ Conservative management to HD

13. What were your reasons for switching modality or treatment (if applicable)

----

14. Which of the following conditions do you have, or you have had before? Show the patient large text options.
    - ❑ Hypertension/ High blood pressure
    - ❑ Diabetes Mellitus
    - ❑ Stroke
    - ❑ Collagen disease
    - ❑ Ischemic heart disease
    - ❑ Congestive heart disease
    - ❑ Gout
    - ❑ Lung disease
    - ❑ Liver disease
    - ❑ Rheumatic tissue disease
    - ❑ Others, please specify..

15. What are the sources of income that support the treatments you mentioned above?
    (e.g., personal income, income from children and relatives, sale of land or assets, government support)

Part 3 Cost information

Patient cost information

16. In this outpatient treatment Patients have medical expenses that must be paid **in addition** to their medical rights.

    Amount ☐☐☐, ☐☐☐, ☐☐☐ Kip

17. **In cases where patients have to pay for accommodation** while waiting for outpatient treatment (e.g. patients coming from other provinces)

    average cost of accommodation this time is " ***for the patient*** **" .** ☐☐☐, ☐☐☐, ☐☐☐ Kip / day by staying for number ☐ of days

18. Round trip travel expenses this time " ***for the patient*** " On average ☐☐☐, ☐☐☐, ☐☐☐ Kip

(In the case of round trip travel with relatives, the average cost per person is calculated **for the patient only**.)

19. Food costs **<u>that are added to</u>** normal life " ***for the patient*** " when receiving treatment at the hospital this time. Average equal to ☐☐☐, ☐☐☐, ☐☐☐ Kip

Costs for relatives / caregivers

20. total number of caregivers of patients who came to care for patients while they were receiving treatment at the hospital ☐☐ person

<u>Caregiver Cost Information Details</u>

Sub-statement

- Fill in the information to calculate the information of each caregiver (e.g. 3 caregivers traveling together, divide by 3 equals 500,000 kip, enter the number "500,000" in each column).
- In the case of no expenses, indicate the number "0".
- In case any item is shared with the patient, such as transportation , accommodation , enter "0". To prevent double counting.
- Round trip is counted as 1 time.

**Cost list/other information**		**Caregiver**		
**section**	**unit**	**Main person**	**The second person**	**The third person**
21. Age of caregiver	year			
22. Relationship with the patient (e.g. father, mother, grandparents)	-			
23. Temporary accommodation rental fee	Kip/day			
24. Number of days of stay in temporary accommodation	day			
25. Round trip travel expenses (Temporary accommodation-Hospital)*	Kip/day			
26. Number of round trips (temporary accommodation-hospital)*	Kip/day<br>Kip/week			
27. Round trip travel expenses (Hospital - Home)*	Kip/day			
28. Number of round trips (Hospital - Home)*	Kip/day<br>Kip/week			
29. Increased food costs for caregivers	Kip/day			
30. Amount lost due to caregiver absenteeism	Kip/day			
31. Other expenses Please specify	Kip/day			

Costs incurred by patients and their relatives outside the hospital

25. In the past 3 months, The patient had previously experienced pain and illness resulting directly or indirectly from kidney disease, requiring special care at home, and causing relatives to take time off work to care for the patient or not

❑ (0) Never.

❑ (1) Number of times, ☐☐ times, duration of illness average ☐☐ day/ time

26. The patient must have relatives take time off work to care for the number ☐ person.

27. Average amount lost due to absenteeism ☐☐☐, ☐☐☐, ☐☐☐ Kip / time / person

28. From item 27 , do the patient's relatives live in the same house as the patient?

❑ (0) No And must pay an average of ☐☐☐, ☐☐☐, ☐☐☐ Kip for round trip travel to the patient's home each time.

❑ (1) Yes.

29. After illness, Do I have to pay for caregivers, such as home care nurses?

❑ (0) No hiring

❑ (1) There is employment, totaling money ☐☐☐, ☐☐☐, ☐☐☐ Kip /month

30. After suffering from end-stage renal disease, the house conditions must be modified to facilitate the patient's daily life, or special equipment must be purchased to reduce problems caused by the illness. Or not

❑ (0) None

❑ (1) Have **(You can answer more than one question .)**

❍a. Purchase amenities such as bed, chair, wheelchair, etc.

specify..

Total amount ☐☐☐, ☐☐☐, ☐☐☐ Kip

❍b. There are changes to the interior of the house, such as house extensions, change the stairs, add handrails and etc. specify..

Total amount ☐☐☐, ☐☐☐, ☐☐☐ Kip

2.2 Indirect costs related to work and activity impairment (WPAI:GH)

The following questions ask about the effect of your health problems on your ability to work and perform your normal activities . " **Health problems"** means problems and illnesses caused by end-stage chronic kidney disease.

***** The following questions*** *Ask about the* *past seven days, excluding today.*

30. In the **past seven days,** how many hours have you been absent from work due to yours health problems?

*** Including the number of hours, you were absent from work due to sick leave, being late for work, leaving work early, and other health-related reasons, but <u>not including the time you participated in this questionnaire.</u>*

Number of ________ hours

31. In the past seven days, how many hours have you been absent from work for other reasons, such as vacation time?

Number of ________ hours

32. past seven days, how many hours have you actually worked ?

Number of ________ hours

33. In the past seven days, how much has your back pain affected your performance while you were at work?

(Think of a day when the amount or type of work you can do is limited, or when you can get less done than you would like, or when you can't work as carefully as you normally would.)

*<u>** If your health problem only affects your work a little, choose a low number and a high number if your health problem affects your work a lot.</u>*

Not affected at all -- > Most affected

**0**	**1**	**2**	**3**	**4**	**5**	**6**	**7**	**8**	**9**	**10**

34. Over the past seven days, how much has your back pain affected your ability to perform daily activities outside of work?

(Routine means the general activities that you do regularly such as housework, shopping, childcare, exercise, study, etc. Think about the time when the number or type of activities you can do are limited and the time when you can complete less than you would like.)

*<u>** If your health problem only affects your work a little, choose a low number and a high number if your health problem affects your work a lot.</u>*

Not affected at all -- > Most affected

**0**	**1**	**2**	**3**	**4**	**5**	**6**	**7**	**8**	**9**	**10**
