# Supplementary material for: Catastrophic health expenditures and economic burden of end-stage kidney disease among patients receiving hemodialysis or conservative care in Lao PDR
Source: PLOS Glob Public Health. 2026 Nov 5;6(11):e0007532. doi: 10.1371/journal.pgph.0007532 (PMC13739169; doi:10.1371/journal.pgph.0007532)
Supplement: Supplementary file 3 [file pgph.0007532.s003.pdf]

Supplementary files

Supplementary 3: Detailed items and unit costs

Direct Medical Costs

Medical cost estimates were based on hospital charges, adjusted using a cost-to-charge ratio of 80%. Table 3 details the treatment services included, grouped by modality (hemodialysis vs. conservative care) and by general/shared services which were applicable to ESKD patients.

General services included one-time costs such as registration fees and diagnostic imaging (e.g., echocardiogram, abdominal X-ray). Costs of treating comorbidities were listed here, and particularly hypertension and type 2 diabetes were common among ESKD patients (Table 1). The annual cost for treating these comorbidities was estimated at 76,346,867 Kip and was assumed to be consistent across treatment groups, given similar baseline of ESKD. Laboratory testing—including CBC, electrolytes, BUN, creatinine, albumin, and urinalysis—was routinely conducted, costing approximately 240,000 Kip per month. Annual lab costs varied by treatment modality due to differing testing frequencies.

For conservative care, the annual direct medical cost was 187,239,638 Kip, covering erythropoietin injections (about 50% of patients) and oral medications (e.g., calcium carbonate, Ferro-B Ca, Amiyu granules (amino acid), folic acid, sodium bicarbonate, hydralazine, and insulin for diabetics). This also covered the cost of laboratory testing, which was performed once every three months on average. Please note that conservative care cohort comprised of patients managed without maintenance hemodialysis (HD) under routine clinical practice.

For HD patients, the initial annual cost was estimated at 210,495,820 Kip, which included a one-time vascular access procedure (2,400,000 Kip) for new patients or those with vein damage/clots. Subsequent annual costs were slightly lower, at 208,095,820 Kip. Dialysis sessions, administered via AVF or DLC, cost between 826,000–892,000 Kip/session, with an average frequency of 2.06 sessions per week among the patients, resulting in monthly costs of 6,791,556–7,334,222 Kip.

Direct Non-Medical Costs

HD households (considering both patients and relatives) incurred significantly higher non-medical costs than those under conservative care, primarily due to more hospital visits, which led to higher expenses for travel and food. The estimated annual non-medical costs were 63,825,795 Kip for HD patients and 30,856,236 Kip for those receiving conservative care.

The number of accompanying relatives was comparable across groups. Interestingly, relatives' food costs were higher than those of patients, because patients were accompanied by multiple family members. However, given that they often travelled together in the same vehicle, this might not elevate overall costs of travelling among them.

As ESKD patients were also commonly presented with multiple comorbidities, there were resources used in response to support such conditions. It is worth mentioning that Additional one-time non-medical expenses included purchases of supportive equipment (average 23,096,164 Kip) and home modifications to accommodate patient needs (average 4,341,026 Kip). However, these costs were included in the sensitivity analysis only.

Opportunity Costs

ESKD management involves frequent hospital visits and often requires family caregivers to take time off work, resulting in opportunity costs. These costs varied by treatment modality due to differences in visit frequency. On average, caregivers of HD patients incurred opportunity costs of approximately 58.2 million Kip/year, compared to 39.7 million Kip/year for caregivers of conservative care patients.

Opportunity costs for patients themselves were not included due to low reported employment rates. Many were unemployed, retired, or engaged in informal work, making income-loss estimates unreliable. Additionally, the patient cohort was generally of older age (Table 1). Inclusion of patient opportunity cost was only performed in the sensitivity analysis.

Table S1 *Detailed cost items among ESKD patients in Lao PDR*

Group/item	Unit Cost (Kip)	95% [CI]	Remark
**Direct Medical Cost**			
**General service/non-specific***			
Registration Book	15,000	NA	Fixed cost
Doctor fee per visit	55,500	33,744–77,256	
Abdominal ultrasound	120,000	41,600–198,400	Applicable only once
X-ray Abdomen	120,000	72,960–167,040	Applicable only once
CT Scan Abdomen	1,868,000	1,029,120–2,706,880	Applicable to operative case (approx. 5% of patients)
Average of physical examination per year	333,400	296,944–369,856	Weighted between USS Abdo, X-ray Abdo, and CT Scan Abdo
Annual cost of treating comorbidities among ESKD patients	76,346,867	53,196,198–99,497,536	See also in table 1 for common comorbidities; IP/OP case visits
**Conservative care[#]**			
Oral medication per month	3,810,000	3,765,312–3,854,688	3 x $CaCO_3$, 3 x FBC, 3 x Amiyu Envelopes, 3 x Folic Acid; 3 x Sodamint; 3 x Hydralazine 25mg; 3 x Isolate 10mg, Insulin
Medicine injection per month	1,920,000	1,292,800–2,547,200	Erythropoietin (EPO) 8 doses/month; patients more than 50% receiving these.
Laboratory tests per month	240,000	192,960–287,040	One time per three months
Annual cost for complication treatment	3,758,371	3,006,697– 4,510,045	
**Hemodialysis (HD)**			
Cost of vascular access	2,400,000	1,459,200– 3,340,800	Included equipment costs

Blood transfusion through a vein in the arm (AVF or AVG) per time	826,000	728,000– 924,000	This estimate was based on standard HD and online HD.
Blood transfusion through Double Lumen Catheter (DLC) per time	892,000	790,080– 993,920	
New cases with Arteriovenous Fistula (AVF)	1,384,000	1,305,600– 1,462,400	
Regular blood transfusion through a vein in the arm (AVF or AVG) per month	6,791,556	5,985,778–7,597,333	Weighted with average HD sessions per month
Regular blood transfusion through Double Lumen Catheter (DLC) per month	7,334,222	6,496,213–8,172,231	Weighted with average HD sessions per month
Laboratory tests per month	240,000	192,960–287,040	Once a month
Annual cost of treating complications among HD patients	3,845,331	3,076,265– 4,614,397	e.g., catheter-related infections
**Direct Non-Medical Cost**			
HD patients			
Cost of round-trip travel to hospital per month	1,326,244	902,034–1,750,455	
Cost of food/beverage per month	346,904	270,224–423,584	
Relatives/caregivers of HD patient			
Adjusted travel cost per month	1,469,862	893,676–2,046,048	In addition to that of patient
Adjusted food/Beverage per month	2,175,806	1,322,890–3,028,721	
Conservative care patients			
Cost of round-trip travels to hospital per month	715,556	467,338–963,773	
Cost of food/beverage per month	153,333	115,220–191,447	
Relatives/caregivers of conservative care patient			
Adjusted travel cost per month	638,634	388,289–888,978	In addition to that of patient
Adjusted food/Beverage per month	1,106,111	672,516–1,539,707	
Special aids/modifications			
Cost of buying additional supporting equipment for patients	23,096,164	13,913,569–32,278,758	Common items include BP Monitor, O2 meter, fingertip test; one-time cost
Cost of space renovation for patients	4,341,026	1,584,234–7,097,817	One-time cost
**Opportunity Cost****			

Household of conservative care patients			Relatives/caregivers
Income loss due to work absence for hospital visits per month	2,138,141	1,244,964– 3,031,318	To accompany patient
Income loss due to work absence per month	955,556	580,978–1,330,133	Outside of hospital visits
Travel cost of relative(s) outside of hospital to deliver care per month	137,778	83,769–191,787	It is rare to hire additional caregiver
Annual informal care costs paid by Conservative Care households	2,646,154	1,608,862–3,683,446	Cost of hiring extra caregiver(s)
Household of HD patients			Relatives/caregivers
Income loss due to work absence for hospital visits per month	3,779,293	1,937,776–5,620,810	To accompany patient
Income loss due to work absence per month	853,333	518,827–1,187,840	Outside of hospital visits
Travel cost of relative(s) outside of hospital to deliver care per month	179,111	108,900–249,323	This is rare
Annual informal care costs paid by HD households	2,646,154	1,608,862–3,683,446	Cost of hiring extra caregiver(s)

Note: *applicable to all patients and not necessarily limited to certain types of KRT modality; #Commonly covered medication (oral), injection (EPO), nutrition, and treatment following symptoms; **the calculation adopted the human capital approach and incorporated multiple variables, including average wage rate, visit frequency, caregiver involvement (proportion and time), and income loss for relatives across HD and conservative care; All costs are reported in 2025 Lao Kip. USD equivalents were calculated using the average 2025 official exchange rate (20,444 Kip = 1 US$) and are presented for international comparison only. Calendar Year Average Consumer Price Index = 251.39; IQR = Interquartile range; 95% CI = 95% Confidence Interval, estimated based on normal approximation method.
